# Differential Accuracy and Concordance of ChatGPT and DeepSeek for Image-Based Questions in Undergraduate Medical Education

**DOI:** 10.1007/s40670-026-02839-9

**Published:** 2026-08-04

**Authors:** Lorraine Silva Requena, Joyce Santana Rizzi, Zilda Maria Tosta Ribeiro, Pedro Tadao Hamamoto Filho, Renato Ferretti

**Affiliations:** 1 Laboratory of Muscle Biology, Institute of Bioscience of Botucatu, Department of Anatomy, São Paulo State University, Botucatu, São Paulo Brazil; 2 Medicine School of Marília, Marília, São Paulo Brazil; 3 Department of Neurosciences and Mental Health, Botucatu Medical School, São Paulo State University, Botucatu, São Paulo Brazil

**Keywords:** Medical education, Large language model, Artificial intelligence, Chatbots, Progress Test, Biological science disciplines

## Abstract

Large language models (LLMs) such as ChatGPT (GPT) and DeepSeek (DS) are increasingly explored in medical education and practice. This study compared the accuracy and concordance of GPT and DS in answering multiple-choice Progress Test questions (2013–2024) across three input formats: text-only, image-only, and full-question (vignette + image). GPT achieved 72.9% overall accuracy versus 65.1% for DS, with moderate inter-model agreement (κ = 0.591). Critically, for both models, full-question accuracy did not exceed text-only accuracy, indicating that the addition of visual content provided no global incremental benefit. Conversely, accuracy dropped significantly in the image-only condition, confirming that isolated visual information was less informative than textual history. Category-specific analyses revealed relative strengths for GPT in skin and body pictures and for DS in microscopic and radiographic images, though these did not translate into an overall multimodal advantage. Questions answered correctly had lower difficulty indices, while discrimination indices did not differ between correct and incorrect responses, and performance was similar across cognitive levels. These findings demonstrate that, under the tested conditions, LLM performance was predominantly text-driven rather than visuospatially integrated. Accordingly, ChatGPT and DeepSeek function as proficient text-based clinical reasoners, but not as effective multimodal diagnostic systems in this educational assessment context.

**Supplementary Information:**

The online version contains supplementary material available at https://doi.org/10.1007/s40670-026-02839-9.

## Introduction

In recent years, advances in artificial intelligence (AI) have resulted in the development of large language models (LLMs) such as ChatGPT (by OpenAI) and DeepSeek (by DeepSeek). These are increasingly being explored as potential tools for medical image analysis [1, 2], detecting drug interactions [3], identifying high-risk patients [2, 4], and the automated coding of medical records [5]. These LLMs have shown promise in processing and generating text-based medical content. However, their performance in interpreting visual information—an ability critical to many fields of medicine—remains limited [6, 7]. The primary reason for this limitation is that these models often lack genuine visuospatial comprehension and depend on statistical correlations between pixel patterns and associated textual labels that have been learned during training [8].

Medical images such as X-rays, computed tomography scans, histopathological biopsies, and magnetic resonance imaging provide essential diagnostic insights that require nuanced interpretation by trained clinicians [9, 10]. Medical experts use their lifetime of structural education and continuous clinical exposure to develop a rich and symbolic understanding of anatomical structures, pathological features, and spatial relationships. This reasoning is grounded in a deep appreciation of the clinical semantics and subtleties that distinguish normal and abnormal findings [10]. By contrast, LLMs are engineered to infer meaning through probabilistic methods that aggregate statistical patterns from large-scale datasets rather than through the explicit encoding of symbolic and visuospatial knowledge [11, 12]. This fundamental difference raises scientific questions regarding the reliability and interpretability of LLM responses in medical contexts and highlights the need to compare models that follow different architectures and training paradigms.

ChatGPT 4.0 represents a generalized approach to LLM development that leverages a vast amount of data and emphasizes chain-of-thought prompting to structure its reasoning processes [8]. Despite these advances, its methodology remains rooted in statistical pattern recognition, which lacks genuine visuospatial comprehension. Conversely, DeepSeek-R1 is characterized by the integration of retrieval-augmented generation techniques and reinforcement learning strategies that aim to ground its responses in factually correct and contextually relevant information [13, 14]. A key difference between these two systems is their approach to visual interpretation. When extended to multimodal tasks, ChatGPT 4.0 converts images into textual descriptions using pretrained vision encoders. Although this method allows the mode to generate plausible textual outputs corresponding to the visual inputs, it does not lead to authentic visuospatial understanding [8]. Consequently, the model’s diagnostic reasoning may overlook the spatial relationships and contextual nuances that are critical in medical imaging. By contrast, DeepSeek-R1 relies on statistical associations drawn from training datasets of images paired with text and lacks the capacity for the true interpretation of visual information [13].

Given the increasing interest in using LLMs in medical education, the educational significance of their performance on image-based questions remains insufficiently defined. Image-based multiple-choice questions require the integration of visual findings with written clinical context, a core component of clinically oriented reasoning and a frequent target of assessment in medical training [6]. Although performance on examination items is not equivalent to real-world diagnostic competence, it can help clarify the extent to which these systems may support multimodal learning, where their limitations remain, and the degree of human oversight necessary before they are implemented in educational settings. Within this framework, Progress Test (PT) questions provide a useful model because they assess clinical knowledge across multiple stages of training and encompass diverse imaging modalities and cognitive demands [15, 16]. Although PT questions were originally designed to assess medical students ‘longitudinal acquisition of clinical knowledge, in the present study they were used as an educationally grounded benchmark to evaluate how LLM respond to items that require interpretation of clinical vignettes, visual information, or their integration. Accordingly, this study evaluated the accuracy and response concordance of ChatGPT and DeepSeek on image-based PT questions and investigation whether performance varied according to input modality, image domain, and cognitive complexity.

## Materials and Methods

An observational, cross-sectional study was conducted utilizing multiple-choice questions derived from a Brazilian interinstitutional Progress Test consortium, administered annually between 2013 and 2024. The Progress Test (PT) is a longitudinal assessment administered simultaneously to all stages of undergraduate medical education to evaluate clinically oriented knowledge under standardized testing conditions [15, 17].

Questions incorporating visual elements were used. Out of the 780 questions available, a subset of 218 questions was selected based on the following inclusion criteria: each question presented a single correct alternative between four options, was structured in a multiple-choice format, and contained a single associated visual content. Single associated visual content means each item included only one visual element linked to the question vignette (e.g., one radiograph, histopathology image, clinical photograph, table, or graph), not multiple figures or mixed visual. The response (correct or incorrect) in each attempt was used as the dependent variable. Students’ actual performance on the questions was used to set their psychometric properties (difficulty and discrimination indices according to the classical test theory) for comparison with the chatbots’ performance.

For the analysis, the independent variables included content area and visual-content category. Visual content was classified in two stages (Tables 1 and 2). First, each eligible item with a single visual element was assigned to an original visual-content category based on the source examination (Table 1). For category-specific analyses, visual features were grouped only when they had comparable visual sources and similar interpretive demands (Table 2). Table 1 summarizes the original item-level visual-content features in the examination questions. Each question had one visual element and was assigned to a single, mutually exclusive feature category based on the source exam’s visual material. Visual formats that appeared only once were grouped into an “Others” category, which included schematic figures, family tree illustrations, network pathway diagrams, and antibiograms. Table 2 presents the broader analytical categories used for category-specific comparions of chatbot performance. The “Others” analytical category included cardiotocograms, partograms, schematic figures, family tree illustration, network pathway diagrams, and antibiograms. Item-level mappings and justifications for each decision are in Supplementary Table S1.

All visual materials were extracted directly from the original examination items and retained in their native layout and resolution as rendered in PDF format. No manual image processing was applied, including but not limited to enhancement, cropping, zooming, contrast modification, or content alteration. For each item, three distinct PDF versions were generated: a full-question version (vignette + answer options + visual content), a text-only version (vignette + answer options, excluding the visual element), and an image-only version (visual element + answer options, excluding the vignette). To minimize heterogeneity in the structure of multimodal input, only items containing a single associated visual element were included.

A complementary framework was used to classify the cognitive demands of each item. Items were classified using an adapted Bloom-based framework reflecting the broader educational level of cognitive processing needed to answer the question: low (remembering/understanding), intermediate (applying/analyzing), and high (evaluating/integrating knowledge to select the best answer) [17]. Cognitive levels were classified exclusively by Dr. ZMTR, a specialist with over 10 years of experience in medical education and assessment. Because a single expert reviewer performed the entire classification, inter-rater agreement procedures were not applicable, and no disagreements required resolution.

All chatbot evaluations were conducted between March and June 2025 using a MacBook computer running MacOS Sequoia 15.5 and the Google Chrome Browser (Version 137.0.7151.120 (Official Build) (arm64)) to access ChatGPT 4.0 and DeepSeek. The latest versions of each model were used during the test. No user-initiated modifications to platform configuration parameters were implemented between experimental runs; however, the possibility of undocumented, provider-side model updates occurring during the study period cannot be definitively ruled out.

To isolate the contribution of each information modality, each item was evaluated under three input conditions: (1) a full question condition (vignette + answer options + visual content), which preserved the original educational format and served as the primary baseline because it best matched authentic exam administration; (2) a text only condition (vignette + answer options, visual content removed), an ablation control estimating the standalone contribution of text; and (3) an image only condition (visual content + answer options, vignette omitted), an ablation control estimating the standalone contribution of visuals. The full question condition was the primary unit of analysis for overall model performance. The text only and image only conditions were used only as sensitivity/control analyses to assess whether performance was driven mainly by textual or visual cues. Comparisons between these two control conditions (e.g., text only vs. image only) were treated as descriptive baselines and not as independent experimental evidence of causal limitations in multimodal reasoning.

For each condition, a new chat session was initiated, and the respective PDF file was uploaded. The chatbot was then prompted to identify the correct alternative (A, B, C, or D) based on the information presented and provide a concise commentary justifying the correct choice and excluding incorrect options. The prompt was “*Based on the information provided in each question and/or accompanying image*,* identify the correct answer (A*,* B*,* C*,* or D). Then*,* for each question*,* provide a brief comment explaining why the correct option is appropriate and why the remaining options are incorrect. Organize your responses in a spreadsheet format with the following columns: Column A (Question number)*,* Column B (Correct answer: A*,* B*,* C*,* or D)*,* and Column C (Commentary on the correct and incorrect options)*.” The responses were compiled into a spreadsheet with three columns: question number, selected answer, and explanatory commentary. After each session, the spreadsheet was downloaded, the results were copied and integrated into a master file, and the chat history was deleted. The browser was then refreshed, and a new chat session was created based on the subsequent experimental condition. An identical prompt was employed across both chatbot platforms and under all experimental input conditions. As the models were accessed via their publicly available web interfaces, generative parameters (e.g., temperature and top-p) were not manually adjusted and therefore remained at the default configurations specified by each platform for the duration of the study.

Categorical data were depicted as percentages with their respective confidence intervals (CIs). Differences between the accuracy and different models were considered significant if the CIs did not overlap. The differences in occurrences of correct answers per group were tested using the Chi-square test. Additionally, the agreement between chatbots was tested using Cohen’s kappa coefficient for paired comparisons. The differences in psychometric properties according to the chatbots and their responses were tested using Student’s *t*-test. We also compared the accuracy and concordance between the chatbots for each group of image content. The analyses were performed using the Statistical Package for the Social Sciences version 24.0 for a MacBook (IBM Corp., Armonk, NY, USA). The level of significance was set at 5% (*p* < 0.05).

## Results

We identified 14 categories of image content, with the most frequent being Table (18.3%), skin pictures (14.7%), and X-ray images (12.8%; Table 1). Regarding the questions’ characteristics, most of them were classified as having a higher cognitive level (Table 1).

**Table 1:** Classification of the included questions according to the content of image features and cognitive complexity levels

Content feature	*N* (%)
Table	40 (18.3)
Skin picture	32 (14.7)
X-ray	28 (12.8)
Histopathology	21 (9.6)
Computed tomography	20 (9.2)
Graph	19 (8.7)
Electrocardiogram	14 (6.4)
Body picture	9 (4.1)
Magnetic resonance imaging	6 (2.8)
Partogram	6 (2.8)
Blood smear	5 (2.3)
Cardiotocogram	5 (2.3)
Ultrasound imaging	4 (1.8)
Other	9 (4.1)
Cognitive level	
Low	22 (10.1)
Middle	88 (40.4)
High	108 (49.5)

**Fig. 1:**
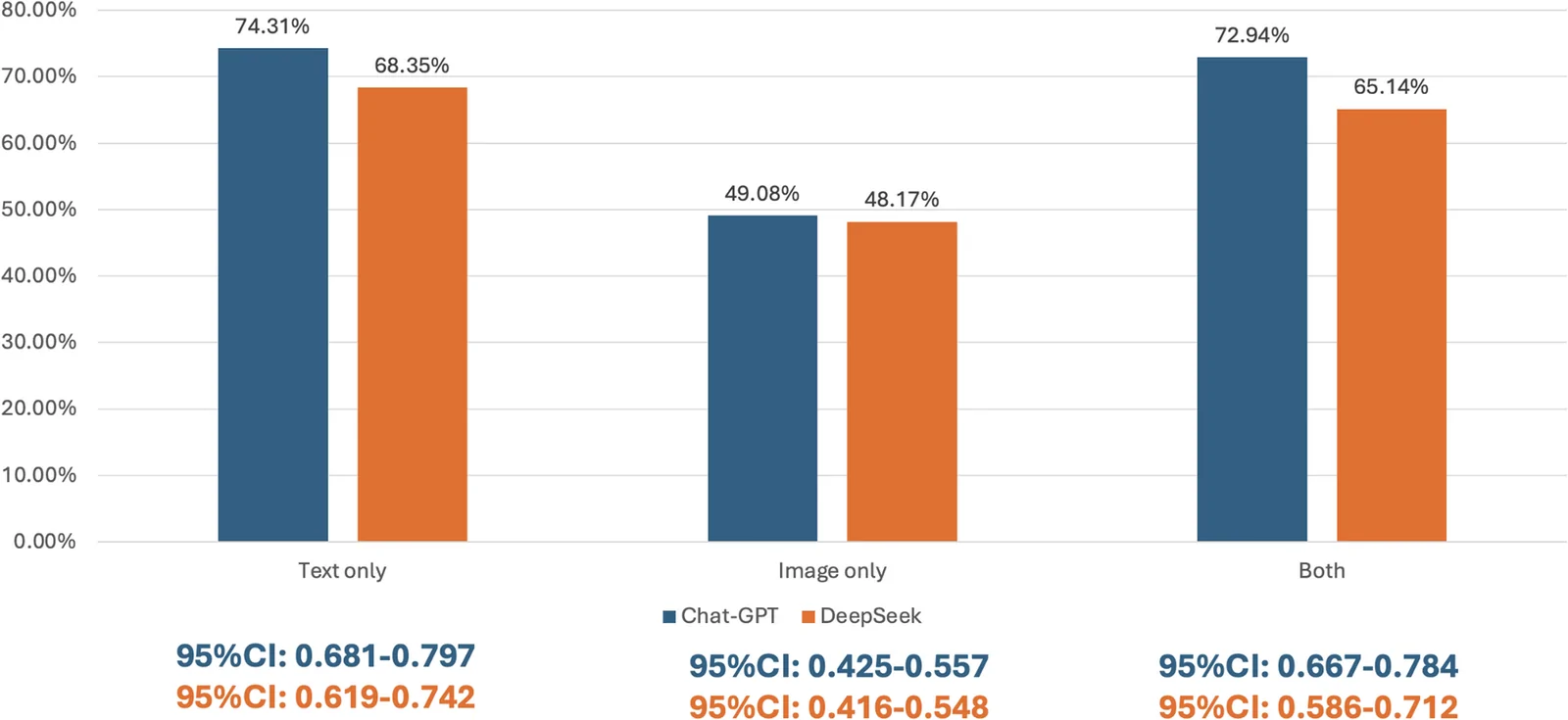
Accuracy (rates of correct answers) of ChatGPT and DeepSeek according to the presentation of text-only, image-only, and full questions. The performance was significantly lower when the chatbots responded to the image-only questions

Across all questions (text and image), the percentage of correct answers was 72.9% (95% CI: 0.667–0.784) for ChatGPT 4.0 and 65.1% (95% CI: 0.586–0.712) for DeepSeek. This percentage did not differ significantly when the chatbots answered the text-only questions. However, there was a significant decrease in accuracy with the image-only questions (Fig. 1). The overall agreement of the chatbots was moderate, with 69.7% of the questions being answered similarly (κ = 0.591, *p* < 0.001).

No difference existed in the performance of the chatbots according to the questions’ cognitive level (Table 2). Irrespective of the cognitive complexity of the questions, the accuracy of both chatbots was lower for image-only questions and similar for text-only and full questions.

**Table 2:** Performance of ChatGPT and DeepSeek on the questions according to their cognitive level considering the full, text-only, and image-only questions

	ChatGPT		DeepSeek	
	Correct answers *N* (%)	*p*-value	Correct answers *N* (%)	*p*-value
	Full question			
Cognitive level				
Low (*n* = 22)	18 (81.8)	0.080	17 (77.3)	0.369
Middle (*n* = 88)	57 (64.8)		54 (61.4)	
High (*n* = 108)	84 (77.8)		71 (65.7)	
	Text only			
Cognitive level				
Low (*n* = 22)	19 (86.4)	0.175	16 (72.7)	0.465
Middle (*n* = 88)	60 (68.2)		56 (63.6)	
High (*n* = 108)	83 (76.9)		77 (71.3)	
	Image only			
Cognitive level				
Low (*n* = 22)	10 (45.5)	0.557	11 (50.0)	0.958
Middle (*n* = 88)	40 (45.5)		43 (48.9)	
High (*n* = 108)	57 (52.8)		51 (47.2)	

**Fig. 2:**
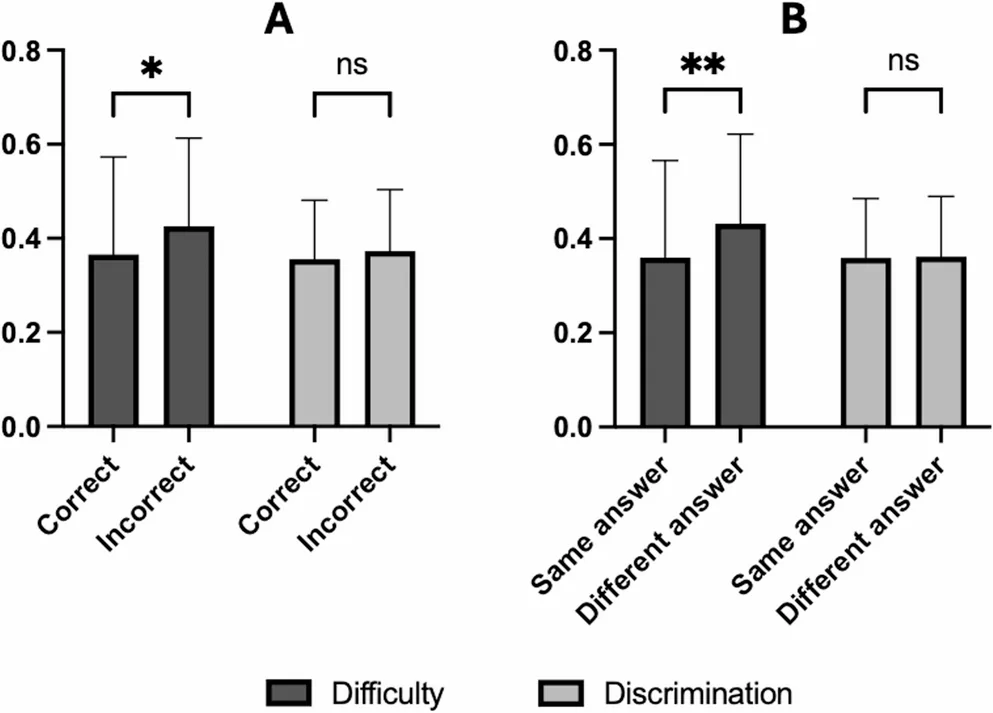
Differences on the psychometric properties (mean and standard deviation of difficulty and discrimination indices) of the questions according to the chatbots’ answers. **A**: Comparison between the correctly and incorrectly answered questions by ChatGPT and DeepSeek. **B**: Comparison between the questions marked similarly and differently by the chatbots. *p = 0.027. **p = 0.007. ns: nonsignificant

The questions answered correctly by the chatbots had lower mean difficulty indices compared with those answered incorrectly (*p* = 0.027). Moreover, when questions were analyzed based on chatbot agreement (i.e., the chatbots marked the same alternative irrespective of being correct), their mean difficulty indices were lower than for the questions marked differently (*p* = 0.007). In contrast, mean discrimination indices showed no significant difference between correctly answered questions and those with chatbots agreement (Fig. 2).

Furthermore, the questions were grouped according to the 14 content features into eight categories: body picture, electrocardiogram, graph, microscopy (blood smear and histopathology), radiographics (X-ray, computed tomography, magnetic resonance imaging, and ultrasound), skin picture, tables, and other (cardiotocogram, partogram, and other).

There were no differences in accuracy rates across the eight groups when testing the full, text-only, and image-only questions for ChatGPT and DeepSeek. The comparison of the accuracy between ChatGPT and DeepSeek for each group was not significantly different, though the absolute rates were slightly higher for ChatGPT. The concordance between the chatbots ranged from 55.0% (other) to 76.9% (microscopy) (Table 3).

**Table 3:** Differential accuracy (rates of correct answers) and agreement between ChatGPT and DeepSeek for each image content group

Group	ChatGPT	DeepSeek	*p*-value*	Concordance^#^	Cohen’s kappa	*p*-value**
Microscopy (*n* = 26)	76.9%	76.9%	1.000	76.9%	0.695	< 0.001
Radiographics (*n* = 58)	75.9%	72.4%	0.675	75.9%	0.673	< 0.001
Skin picture (*n* = 32)	90.6%	71.9%	0.055	71.9%	0.586	< 0.001
Electrocardiogram (*n* = 14)	71.4%	50.0%	0.246	71.4%	0.600	< 0.001
Table (*n* = 40)	62.5%	65.0%	0.818	67.5%	0.552	< 0.001
Graph (*n* = 19)	68.4%	52.6%	0.317	63.2%	0.511	< 0.001
Body picture (*n* = 9)	88.9%	66.7%	0.259	55.6%	0.368	0.070
Others (*n* = 20)	50.0%	40.0%	0.525	55.0%	0.416	< 0.001

* Refers to the difference of accuracy between the chatbots

^#^ Refers to the percentage in which both chatbots answered the same questions correctly

** Refers to the Cohen’s kappa value

In summary, ChatGPT and DeepSeek had an overall accuracy of 70% for image-based multiple-choice questions, with ChatGPT being slightly superior on marking the correct choice. Accuracy was significantly lower when the chatbots did not have access to the question vignettes. For ChatGPT, a higher percentage of correct answers was observed for questions based on skin and body pictures, and a lower percentage was observed for tables and graphs. For DeepSeek, a higher percentage of correct answers was observed for microscopic and radiographic images, and a lower percentage was observed for electrocardiogram, tables, and graphs. Questions with incorrect or discrepant answers between the chatbots had higher difficulty indices for undergraduate medical students. The concordance between the chatbots was moderate, with a higher Cohen’s kappa for the microscopic and radiographic images.

## Discussion

Large language models such as ChatGPT and DeepSeek performed similar to mid-level medical students when addressing integrated text–image examination questions [18–20]. However, a critical finding that qualifies this interpretation is that, for both models, performance in the full‑question condition did not differ significantly from the text‑only condition (Fig. 1). This statistical equivalence indicates that, across the entire dataset, the inclusion of visual material did not confer an incremental benefit in terms of diagnostic accuracy. In other words, model performance on these items was primarily driven by comprehension of the textual clinical vignette rather than by substantive visuospatial processing or integration of the associated images. The marked decrease in accuracy in the image‑only condition further substantiates this interpretation: when the textual context was removed, performance declined sharply, demonstrating that the models are strongly dependent on linguistic cues. Taken together, these findings indicate that, at a global level, the evaluated LLMs are more appropriately characterized as text‑based clinical reasoners than as genuinely multimodal diagnostic systems.

When comparing the control conditions, accuracy was lower in the image-only condition than in the text-only condition. However, this comparison contrasts two unimodal ablation conditions rather than the primary multimodal comparison and should not be interpreted as stand-alone evidence of a fundamental limitation in multimodal reasoning. Instead, it indicates that, in this dataset, images presented without the accompanying clinical vignette yielded lower accuracy than textual vignettes presented without images. This pattern may help explain why performance in the full-question condition was similar to that observed in the text-only condition.

Crucially, the overall similarity between full-question and text-only accuracies indicates that, across the dataset, models are driven almost entirely by the textual vignette rather than by effective multimodal integration of visual content. The visual component neither systematically enhanced nor degraded performance when the vignette was present, suggesting that the models’ primary reasoning strategy relies on linguistic cues, with visual inputs contributing little incremental information at the global level.

ChatGPT was slightly better than DeepSeek for integrated text–image diagnostics (especially with body and skin pictures), whereas DeepSeek excelled in microscopic and radiographic images. In our assessment, the dataset consisted of higher-order questions, with nearly 50% of the items requiring application, analysis, or reasoning rather than simple factual recall. Overall, the study design, which categorized tasks into text-only, image-only, and integrated modalities, emphasized the efficacy of LLMs and emphasized their potential pedagogical applicability under specific multimodal conditions, while simultaneously delineating critical constraints. The employed methodologies provided evidence that the effectiveness of LLMs was significantly dependent on the input format, with text-based contexts serving as their most reliable capability. However, reasoning across multiple modalities remained an area of continued progression.

The comparable performance observed between the full-question and text-only conditions, coupled with the pronounced decrease in accuracy in the image-only condition, indicates that the models’ predominant reasoning strategy is text-dependent. Accordingly, the reduction in image-only accuracy does not primarily reflect a deficit in multimodal integration per se, but instead suggests that the visual modality contributes minimal additional information beyond what is already available in the textual vignette. This pattern is consistent with prior evidence that contemporary vision–language models frequently exploit textual shortcuts rather than engaging in genuine visuospatial reasoning [21, 22]. The moderate agreement between the models (k ~ 0.6) suggested convergent but not identical reasoning strategies, reflecting differences in the training data and model design [23, 24]. In text-based contexts, natural language processing performs better than multimodal reasoning owing to its advanced development. ChatGPT uses reinforcement learning with human feedback and fine-tuning to enhance reasoning and logical skills [20]. Although DeepSeek uses similar architectures, it differs in pretraining data and objectives, possibly leading to lower accuracy. These differences emphasize the importance of model size and training strategy for performance [24]. Our findings suggested that, while LLMs demonstrate promising capabilities, further refinement of multimodal learning and domain-specific adaptation could be helpful for reliable application in education and medical practice.

The Progress Test, oriented around clinic-based multiple-choice questions, is designed to ensure stringent yearly cross-institutional evaluation [15, 17]. Developed by a collaborative group of universities, this examination assesses medical students from their initial to final years in medical schools in Brazil [15, 17, 20]. The examination proficiently assesses the advancement of students’ competencies and preparedness for clinical practice by incorporating fundamental concepts from basic medical sciences [15, 23]. Although prior investigations have indicated that LLMs can achieve performance levels comparable to those of intermediate medical trainees on examination-style questions, the present study advances this literature by demonstrating that such performance is highly dependent on the informational format of the test item, the specific nature of the visual content, and the underlying psychometric properties of the question [15, 25]. This differentiation is pertinent to medical education, as it implies that LLMs may be more appropriately deployed for guided review and formative support in selected multimodal settings, rather than for unsupervised interpretation of items in which accurate responses are critically dependent on image-based information. Therefore, despite the need for advancements in AI capabilities and the extensive data processed by generative AI, its effective use and application in medical education continues to rely on careful guidance from educators to ensure precise and meaningful learning for medical students [16].

The type of image content influenced performance. ChatGPT performed better in tasks involving skin and body pictures, likely benefiting from the exposure to visual datasets and reinforcement learning with human feedback [8, 26]. By contrast, DeepSeek achieved higher accuracy in microscopy and radiographic images, possibly because of the differential emphasis on structured biomedical data during pretraining. These findings align with previous evidence that multimodal models exhibit domain-specific variability depending on the image modality tested [21, 27–29]. The visual stimuli employed in the present study did not impose uniform cognitive demands. In contrast to clinical photographs, microscopy images, or radiographic studies, tables and graphs primarily encode structured quantitative information rather than predominantly anatomical or morphological features. Their interpretation necessitates the extraction of symbolic and numerical relationships, identification of trends, and integration of these data with the accompanying vignette. These additional cognitive requirements may account for the comparatively lower performance observed for both models in these stimulus categories.

Evaluation beyond memorization required the integration of clinical knowledge with the interpretation of visual content, such as tables, patients, or radiographic images [30]. Cognitive complexity did not alter performance. This emphasized the importance of the contextual integration between text and images for higher-order reasoning [6]. The observation that correctly answered questions had lower difficulty indices indicated that the current levels of question discrimination and cognitive challenge were limited, as previously observed [27], whereas the questions’ capacity to discriminate among higher difficulty levels was limited. This pattern mirrors earlier findings in AI-assisted assessments, where models performed reliably on recall and integrated tasks but less so for nuanced diagnostic challenges [31, 32].

The moderate concordance between ChatGPT and DeepSeek across categories, which was the highest for microscopy and radiographic images and lowest for body pictures, highlighted the partial overlap in model reasoning. Although both LLM systems have demonstrated utility in processing multimodal content, their differences suggest complementary strengths that can be leveraged in medical education [12, 31, 33]. In particular, combining LLMs for textual reasoning with dedicated vision-based models, such as convolutional neural networks, may yield synergistic improvements in medical training applications [33].

From an educational standpoint, these findings support the positioning of LLMs as adjunctive instructional resources rather than as autonomous evaluative systems in image-based medical education. Their comparatively superior performance when provided with both textual and visual inputs suggests that they may be particularly valuable for structured review, the provision of formative feedback, and the facilitation of discussion around clinically contextualized questions [32]. Conversely, the pronounced reduction in accuracy under image-only conditions indicates that students should be explicitly cautioned against using these models for standalone interpretation of visual materials, especially when the task relies heavily on radiographic, graphical, or tabular reasoning. Accordingly, educators should conceptualize and present LLM use as a supervised, multimodal learning aid, rather than as a replacement for expert instruction or formal training in visual diagnostic skills.

This study had some limitations. Variables such as internet speed, online traffic, specific versions of the chatbots used, and potential processing delays might have affected the AIs’ responses. Non-English questions might have resulted in less accurate responses owing to possible misinterpretations compared with the English language content. As discussed in the previous section, the comparable performance between the image-only and full-case conditions in specific categories likely reflects category-specific redundancy of visual cues or pathognomonic features, rather than evidencing that clinical vignettes are broadly unnecessary. This interpretation is expanded with detailed hypotheses in the Discussion above. Additionally, because the Progress Test is based on clinical cases in the Brazilian context, the chatbots’ performance may differ on international certification exams. Our findings should also be interpreted in the context of the specific large language models available during the study period, namely ChatGPT 4.0 and DeepSeek, which were evaluated between March and June 2025, as the rapid and ongoing evolution of LLM architectures and training regimes may affect the reproducibility and generalizability of these results over time. Finally, our findings cannot be generalized to other medical disciplines because chatbots’ knowledge bases are continuously updated through user interactions and feedback.

## Conclusion

ChatGPT and DeepSeek achieved accuracies comparable to mid-level medical students when textual context was available. Full-question accuracy did not exceed text-only accuracy, indicating negligible incremental value from images globally, whereas image-only accuracy dropped sharply. These patterns confirm that performance was text-driven rather than visuospatially integrated. Thus, under the tested conditions, these LLMs function as proficient text-based reasoners rather than effective multimodal diagnostic systems. Future studies should systematically quantify image value using balanced, clinically authentic cases and standardized protocols, incorporating psychometric indices to guide educational applications.

## Supplementary Information

Supplementary Material 1.

## References

[1] Lu MY, Chen B, Williamson DFK, Chen RJ, Zhao M, Chow AK, et al. A multimodal generative ai copilot for human pathology. Nat Springer Sci Bus Media LLC. 2024. 10.1038/s41586-024-07618-3. PMC11464372 38866050

[2] Ali M, Benfante V, Basirinia G, Alongi P, Sperandeo A, Quattrocchi A, et al. Applications of artificial intelligence, deep learning, and machine learning to support the analysis of microscopic images of cells and tissues. J. Imaging. Multidisciplinary Digital Publishing Institute (MDPI); 2025. 10.3390/jimaging11020059. PMC11856378 39997561

[3] Yi HC, You ZH, Huang DS, Li X, Jiang TH, Li LP. A Deep Learning Framework for Robust and Accurate Prediction of ncRNA-Protein Interactions Using Evolutionary Information. Mol Ther Nucleic Acids Cell Press. 2018;11:337–44. 10.1016/j.omtn.2018.03.001. PMC5992449 29858068

[4] Saeidnia HR, Firuzpour F, Kozak M, et al. Advancing cancer diagnosis and treatment: integrating image analysis and AI algorithms for enhanced clinical practice. Artif Intell Rev. 2025;58:105. 10.1007/s10462-025-11117-w

[5] Akpan IJ, Kobara YM, Owolabi J, Akpan AA, Offodile OF. Conversational and generative artificial intelligence and human–chatbot interaction in education and research. Int Transact Oper Res. John Wiley and Sons Inc; 2024. 10.1111/itor.13522

[6] Sau SGDSRKGLAJMSAFTRRVGSJ. Accuracy and quality of ChatGPT-4o and Google Gemini performance on image-based neurosurgery board questions. Neurosurg Rev. 2025;25:320. 10.1007/s10143-025-03472-7 40131528

[7] Sagoo MG, Vorstenbosch MATM, Bazira PJ, Ellis H, Kambouri M, Owen C. Online assessment of applied anatomy knowledge: the effect of images on medical students’ performance. Anat Sci Educ John Wiley Sons Inc. 2021;14:342–51. 10.1002/ase.1965. 32289198

[8] Achiam J, Adler S, Agarwal S, Ahmad L, Akkaya I et al. GPT-4 Technical Report. 2023. https://arxiv.org/abs/2303.08774

[9] American Educational Research Association APA. American Educational Research Association APA, & National Council on Measurement in Education. Standards for educational and psychological testing. Washington, DC: Washington, DC:: American Educational Research Association; 2014.

[10] Anderson LW, Krathwohl DR, Airasian PW, et al. A taxonomy for learning, teaching, and assessing: a revision of Bloom’s Taxonomy of Educational Objectives. New York: New York: Addison Wesley Longman; 2001.

[11] Eysenbach G. The Role of ChatGPT, Generative language models, and artificial intelligence in medical education: a conversation with chatgpt and a call for papers. JMIR Med. Educ. JMIR Publications Inc.; 2023. 10.2196/46885. PMC10028514 36863937

[12] Sallam M. ChatGPT utility in healthcare education, research, and practice: systematic review on the promising perspectives and valid concerns. Healthcare (Switzerland). MDPI; 2023. 10.3390/healthcare11060887 PMC10048148 36981544

[13] França Pedro RVP, Sá S, Alves-Costa, Poliana C, de Viola AF, Costa SérgioS, Bruno F, de Souza JoãoDS, de Almeida JoãoOB, Diniz, Cecilia CC. Ribeiro. MarIA - DeepSeek: Uma Proposta de Assistente por Modelo Amplo de Linguagem para Agentes Comunitários de Saúde. Anais do XXV Simpósio Brasileiro de Computação Aplicada à Saúde, Porto Alegre/RS, 2025. SBC, 2025, pp. 305–316.

[14] Brodeur PG, Buckley TA, Kanjee Z, Goh E, Ling E, Bin, Jain P et al. Superhuman performance of a large language model on the reasoning tasks of a physician. 2026;392:524–527. 10.1126/science.adz4433 42060751

[15] Bicudo AM, Hamamoto Filho PT, Abbade JF, Hafner M, de LMB, Maffei CML. Teste de progresso em consórcios para todas as escolas médicas do Brasil. Rev Bras Educ Med FapUNIFESP (SciELO). 2019;43:151–6. 10.1590/1981-52712015v43n4rb20190018.

[16] Schuwirth LWT, van der Vleuten CPM. The use of progress testing. Perspect. Med. Educ. Bohn Stafleu van Loghum; 2012. pp. 24–30. 10.1007/s40037-012-0007-2 PMC3540387 23316456

[17] Hamamoto Filho PT, Silva E, Ribeiro ZMT, Hafner M, de LMB, Cecilio-Fernandes D, Bicudo AM. Relationships between Bloom’s taxonomy, judges’ estimation of item difficulty and psychometric properties of items from a progress test: A prospective observational study. Sao Paulo Medical Journal. Associacao Paulista de Med. 2020;138:33–9. 10.1590/1516-3180.2019.0459.r1.19112019. PMC9673841 32321103

[18] Albano MG, Cavallo F, Hoogenboom R, Magni F, Majoor G, Manenti F, et al. An international comparison of knowledge levels of medical students: The Maastricht progress test. Med Educ Blackwell Publishing Ltd. 1996;30:239–45. 10.1111/j.1365-2923.1996.tb00824.x. 8949534

[19] Finucane P, Flannery D, Keane D, Norman G. Cross-institutional progress testing: Feasibility and value to a new medical school. Med Educ. 2010;44:184–6. 10.1111/j.1365-2923.2009.03567.x. 20059672

[20] Romaní-Romaní F, Gutiérrez C. Trends in progress test performance of medical students at a university in Peru. PLoS One Public Libr Sci. 2025;20(8):e0330029. 10.1371/journal.pone.0330029 PMC12324671 40763293

[21] Jin Q, Chen F, Zhou Y, Xu Z, Cheung JM, Chen R, et al. Hidden flaws behind expert-level accuracy of multimodal GPT-4 vision in medicine. NPJ Digit Med Nat Res. 2024;7:190. 10.1038/s41746-024-01185-7 PMC11266508 39043988

[22] Wang H, Wu WZ, Dou Z, He L, Yang L. Performance and exploration of ChatGPT in medical examination, records and education in Chinese: Pave the way for medical AI. Int J Med Inf Elsevier Irel Ltd. 2023;177:105173. 10.1016/j.ijmedinf.2023.105173 37549499

[23] Kipp M, From. GPT-3.5 to GPT-4.o: A Leap in AI’s Medical Exam Performance. Inform MDPI AG. 2024;15:543. 10.3390/info15090543.

[24] Zhao R et al. Maximum entropy population-based training for zero-shot human-ai coordination. In: Proceedings of the AAAI Conference on Artificial Intelligence. 2023. pp. 6145-6153. https://arxiv.org/abs/2112.11701

[25] Ouyang, Long, et al. "Training language models to follow instructions with human feedback. (2022). https://arxiv.org.abs.2203.02155

[26] Hamamoto Filho PT, Hashimoto M, Lima AR, de Diehl A, Costa LA, Rehder NT. Reliability across content areas in progress tests assessing medical knowledge: a Brazilian cross-sectional study with implications for medical education assessments. Sao Paulo Med J Associacao Paulista de Med. 2024;142(6):e:2023291. 10.1590/1516-3180.2023.0291.R1.13052024 PMC11251435 39016382

[27] Ferretti R, Rizzi JS, Requena LS, Bicudo AM, Hamamoto Filho PT. Factors associated with lower performance of artificial intelligence on answering undergraduate medical education multiple-choice questions. Med Sci Educ. Springer; 2025; 10.1007/s40670-025-02426-4 PMC12532555 41112890

[28] Lu, Haoyu, et al. DeepSeek-VL: Towards real-world vision-language understanding, 2024. https://arxiv.org/abs/2403.05525

[29] Santana Rizzi J, Silva Requena L, Bicudo AM, Hamamoto Filho PT, Ferretti R. Chatbot underperformance in biology and image-based questions in medical education. J CME. 2025;14(1):2596550. 10.1080/28338073.2025.2596550 PMC12679838 41356263

[30] Al-Thani SN, Anjum S, Bhutta ZA, Bashir S, Majeed MA, Khan AS, et al. Comparative performance of ChatGPT, Gemini, and final-year emergency medicine clerkship students in answering multiple-choice questions: implications for the use of AI in medical education. Int J Emerg Med BioMed Cent Ltd. 2025;18:146. 10.1186/s12245-025-00949-6 PMC12329864 40775272

[31] Sallam M, Al-Salahat K, Eid H, Egger J, Puladi B. Human versus Artificial Intelligence: ChatGPT-4 Outperforming Bing, Bard, ChatGPT-3.5 and humans in clinical chemistry multiple-choice questions. Adv Med Educ Pract. 2024;15:857–71. 10.2147/AMEP.S479801. PMC11421444 39319062

[32] Al-Khater KMK. Comparative assessment of three AI platforms in answering USMLE Step 1 anatomy questions or identifying anatomical structures on radiographs. Clin Anat. John Wiley and Sons Inc; 2024; 10.1002/ca.24243 39558670

[33] Esteva A, Chou K, Yeung S, Naik N, Madani A, Mottaghi A, et al. Deep learning-enabled medical computer vision. NPJ Digit Med Nat Res. 2021. 10.1038/s41746-020-00376-2. PMC7794558 33420381

