# Supplementary material for: Differential Accuracy and Concordance of ChatGPT and DeepSeek for Image-Based Questions in Undergraduate Medical Education
Source: Med Sci Educ. 2026 Aug 4;36(6):3009–17. doi: 10.1007/s40670-026-02839-9 (PMC13759941; doi:10.1007/s40670-026-02839-9)
Supplement: Supplementary file 1 [file 40670_2026_2839_MOESM1_ESM.docx]

**Supplementary Table S1.** Mapping of original visual-content features to analytical categories and rationale for grouping.

| **Original visual-content feature (Table 1)** | **Analytical category (Table 2)** | **Rationale for classification or grouping** |
| --- | --- | --- |
| Histopathology | Microscopy | Requires morphological recognition and interpretation at the cellular or microscopic level. |
| Blood smear |  |  |
| X-ray | Radiographics | Despite their different physical principles, all are clinical imaging modalities used to interpret internal anatomical structures and pathological findings. |
| Computed tomography (CT) |  |  |
| Magnetic resonance imaging (MRI) |  |  |
| Ultrasound imaging (US) |  |  |
| Skin picture | Skin picture | Depicts macroscopic, externally visible clinical findings assessed by visual inspection. Furthermore, skin photographs primarily required assessment of lesion morphology, color, borders, texture, and distribution. |
| Body picture | Body picture | Body photographs were used to evaluate broader regional or whole-body features, such as external configuration, posture, deformities, or other macroscopic findings. |
| Electrocardiogram | Electrocardiograms | A standardized clinical waveform tracing requiring interpretation of temporal patterns, intervals, waveform morphology, amplitude relationships, and clinically relevant thresholds. |
| Table | Tables | Requires extraction, comparison, and integration of discrete information organized in rows and columns. |
| Graph | Graphs | Requires spatial interpretation of relationships among variables, including trends, curves, axes, and distributions. |
| Cardiotocogram | Others | Visual format with a specific clinical interpretation framework that is not directly equivalent to electrocardiograms, tables or graphs. |
| Partogram |  |  |
| Schematic figure (Others) |  |  |
| Family tree (Others) |  |  |
| Network pathway diagram (Others) |  |  |
| Antibiogram (Others)  Anatomical images (Others) |  |  |
